# The role of DNA demethylation in liver to pancreas transdifferentiation

**DOI:** 10.1186/s13287-022-03159-6

**Published:** 2022-09-16

**Authors:** Adi Har-Zahav, Daniela Lixandru, David Cheishvili, Ioan Valentin Matei, Ioana Raluca Florea, Veronica Madalina Aspritoiu, Inna Blus-Kadosh, Irit Meivar-Levy, Andreea Madalina Serban, Irinel Popescu, Moshe Szyf, Sarah Ferber, Simona Olimpia Dima

**Affiliations:** 1 Institute of Gastroenterology, Hepatology and Nutrition, Schneider Children’s Medical Center of Israel, Petach Tikva, Israel; 2 Sackler School of Medicine, Felsenstein Medical Research Center, Tel Aviv University, Tel Aviv, Israel; 3 The Sheba Regenerative Medicine, Stem Cell and Tissue Engineering Center, Sheba Medical Center, 5262100 Tel-Hashomer, Israel; 4 University of Medicine and Pharmacy ‘Carol Davila’, 050474 Bucharest, Romania; 5 Department of Oncology, McGill University, Montreal, QC Canada; 6 HKG Epitherapeutics, Honk Kong, China; 7 Dia-Cure, Acad. Nicolae Cajal Institute of Medical Scientific Research, Titu Maiorescu University, 040441 Bucharest, Romania; 8 Systems Biology of Aging Group, Institute of Biochemistry of the Romanian Academy, 060031 Bucharest, Romania; 9 Biology and Biochemistry Department, Faculty of Biology, Bucharest University, 050095 București, Romania; 10 Orgenesis Ltd, 7414002 Ness Ziona, Israel; 11 Center of Excelence in Translational Medicine, Fundeni Clinical Institute, 022328 Bucharest, Romania; 12 Department of Human Genetics, Sackler School of Medicine, Tel Aviv University, 6997801 Tel Aviv, Israel

**Keywords:** Transdifferentiation, Liver, Pancreas, Pancreatic transcription factors, Epigenetic modifications, DNA methylation, Cell replacement therapy for diabetes

## Abstract

**Background:**

Insulin producing cells generated by liver cell transdifferentiation, could serve as an attractive source for regenerative medicine. The present study assesses the relationship between DNA methylation pTFs induced liver to pancreas transdifferentiation.

**Results:**

The transdifferentiation process is associated with DNA demethylation, mainly at gene regulatory sites, and with increased expression of these genes. Active inhibition of DNA methylation promotes the pancreatic transcription factor-induced transdifferentiation process, supporting a causal role for DNA demethylation in this process.

**Conclusions:**

Transdifferentiation is associated with global DNA hypomethylation, and with increased expression of specific demethylated genes. A combination of epigenetic modulators may be used to increase chromatin accessibility of the pancreatic transcription factors, thus promoting the efficiency of the developmental process.

**Supplementary Information:**

The online version contains supplementary material available at 10.1186/s13287-022-03159-6.

## Introduction

Numerous studies suggest that adult cell differentiation fate can be reprogrammed by overexpression of selected transcription factors, usually a subset of those required for normal development of the relevant cell type (reviewed in [1]). Using this approach, ectopically expressed pancreatic specific transcription factors were shown to trigger to a comprehensive and irreversible process of transdifferentiation (TD) of liver into pancreatic cells. Ectopic pancreatic Transcription Factors (pTFs) were hypothesized to induce heritable, epigenetic alterations in liver cells, that in turn, cause broad alterations in the landscape of gene expression, thus altering cellular identity [2–5]. However, ectopic pTF expression has proven obligatory but insufficient for activating the pancreatic lineage in liver cells, as most human liver cells resisted the pTF-induced TD process, while just a minority of cells trans-differentiated in response to ectopic pTFs expression delivered by recombinant adenoviruses [6, 7]. What determines the propensity of certain liver cells to undergo TD in response to ectopic pTF? It suggested that a particular status of intracellular signaling pathways and a permissive epigenetic landscape of the host cells define whether pTFs induce TD [8].

In a previous study, we identified crucial factors that differentiate liver-derived cells displaying a TD-propensity, which undergo pTF-induced reprogramming into pancreatic IPCs, from those that fail to undergo TD [7]. Cells that undergo TD display an active Wnt signaling activity; however, Wnt signaling is insufficient on its own to endow recalcitrant cells with reprogramming capacity. TD requires, in addition to an active Wnt signaling pathway, treatment with a histone deacetylase inhibitor (HDACi) [7]. This suggests that restrictions imposed by repressive chromatin organization in most liver cells are responsible for resistance to pTFs-induced TD.

Indeed, it has been suggested that epigenetic modulators, such as histone modifications and DNA methylation, that play a crucial role in organ development and lineage commitment may also affect the process of pTFs-induced adult cells reprogramming or TD [8]. The present study aims to analyze the relative role of DNA methylation in the process of liver-to-pancreas TD.

DNA methylation is an epigenetic process by which methyl groups are transferred from S-adenosylmethionine to the C5 position of the cytosine to form 5-methylcytosine via DNA methyltransferases (DNMT). In mammals, DNA methylation is mostly found in CpG dinucleotides [9]. Most CpG islands located in the promoter region of active genes are unmethylated, whereas hypermethylation of CpG islands are associated with gene silencing [10]. Transcription silencing caused by DNA methylation is achieved either directly by inhibiting the binding of transcription factors to DNA, or indirectly by recruiting other chromatin-modifying proteins, such as DNA methylation binding proteins and histone deacetylases (HDACs) [11].

A recent study suggests a role for DNA methylation status in β-cells neogenesis, and that DNA methylation is a barrier in β cell regeneration in the adulthood [12]. Moreover, DNA methylation could be also involved in mediating the pathophysiological effects of β cell deficiency in diabetes in the adult pancreas [12]

The present study analyzes the role of DNA methylation of host liver cells in the process of liver-to-pancreas TD. The study suggests that pTF-induced liver-to-pancreas TD is associated with gradual induction of DNA hypomethylation. In addition, artificial induction of DNA hypomethylation of human liver cells either by 5-AZA or by diminishing the DMNT1 levels, promote the pTF-induced liver-to-pancreas TD process, suggesting a causal role for the loss of DNA methylation in TD.

## Methods

### Human liver tissues and cultures

Liver tissues were used with the approval of the Institutional Review Boards of Sheba Medical Center and Fundeni Clinical Institute, after obtaining informed consent of the donors or of legal representatives. Tissue processing, isolation, and the maintenance of cell cultures were previously described [13]. Hepatic wedges of 1.5–2 g were used for generating adherent cultures of liver cells. Cells were seeded at a density of 4000 cells/cm^2^, in DMEM with 1 g/L glucose supplemented with 2 mM L-glutamine, 10% FBS and 100U/mL penicillin/100 µg/mL streptomycin/250 ng/mL amphotericin (Thermo Fisher Scientific, MA, USA). Cells were routinely sub-cultured at 80% confluence. The cell population was expanded up to passage 12 in vitro.

### Induction of TD *in-vitro*

Cells were seeded at a density of 10,000 cells/cm^2^, in TD media consisting of low glucose DMEM supplemented with 2 mM L-glutamine, 10% FBS, 10 mM nicotinamide (Sigma-Aldrich), 20 ng/mL epidermal growth factor (PeproTech), 5 nM exendin-4 (Sigma-Aldrich), and antibiotics, 100U/mL penicillin/100 µg/mL streptomycin/ 250 ng/mL amphotericin (Thermo Fisher Scientific, MA, USA).

The cells were infected with recombinant adenoviruses as follows: 1000 MOI Ad-*PDX-1* and 250 MOI Ad-*NEUROD1;* Ad-CMV-β-Gal was used as infection control, as described previously [7, 14]. After 48 h, the cells were harvested, counted, infected with 50 MOI of Ad-*MAFA* and re-plated as above. Seventy-two hours later, the cells were analyzed for gene expression as described previously [7, 14].

### Soluble factors

Suberoylanilide hydroxamic acid (SAHA; Sigma) was added at a final concentration of 1 µM, 5-Azacytidine (5-AZA, Sigma) was added at final concentrations of 1–4 µM, lithium chloride (LiCl, Sigma) was added at a final concentration of 10 µM, as indicated previously [7].

### Gene expression analysis

RNA was extracted from cells and reverse-transcribed as previously described [4]. Relative expression of the indicated genes was measured by Real-Time PCR using SYBR Green Master Mix (Thermo Fisher Scientific), with *TBP* as reference. Amplification primers are detailed in Additional file 2: Table S1.

### DNA isolation

Total genomic DNA was isolated using DNeasy Blood & Tissue Kit (QIAGEN cat. no. 69504 or 69506) according to the manufacturer's instructions.

### DNA methylation data processing

Genomic DNA from the cells was quantified using Picogreen protocol (Quant-iTTM PicoGreen dsDNA Products, Invitrogen, P-7589) and read on a Spectra-MAX GeminiXS Spectrophotometer. Bisulfite conversion of 500 ng of genomic DNA was performed using the EZ-96 DNA Methylation-GOLD Kit (Zymo Research, Irvine, CA, USA). The Illumina Methylation 450 K kit (San Diego, California, USA) was used for the microarray experiment as described by the manufacturer’s protocol, except that 8 uL of bisulfite converted template was utilized to initiate the amplification step. The Illumina hybridization oven was used for incubating amplified DNA (37 °C) and for BeadChips hybridization (48 °C).

A Hybex incubator was used for the fragmentation (37 °C) and denaturation (95 °C) steps. The X-stain step was carried out in a Tecan Freedom evo robot with a Te-Flow module (Tecan Group Ltd, Mannedorf, Switzerland). Arrays were scanned in an Illumina iScan Reader.

The raw data obtained from the Illumina 450 K and EPIC arrays were processed from the IDAT files through to normalization with BMIQ [15] using the ChAMP [16] pipeline, batch correction for technical replication dataset using ComBat [17] and all subsequent analyses were performed with the R statistical software v3.2.1.

Quality control of the array data included removal of probes for which no sample passed a 0.01 detection *P*-value threshold, and filtering probes with a bead count less than 3.

### DNMT1 ShRNA

shDNMT1 construct for lentivirus infection was purchased from open biosystems, and preparation of viral particles was previously described [18]. Primary human liver cells were seeded at 50–70% confluent on 10 cm plates. The cells were infected with 3 mL lentivirus using 8 ng/ml polybrene carrier (Sigma) over-night. Once cells were more than 90% confluent, they were detached with trypsin–EDTA (0.25%) and replated at a 1:2 dilution. The infected cells were kept under puromycin selection for two weeks as previously described [18], and the infection efficiency was measured by GFP content, DNMT1 RNA and protein levels.

### Protein purification

Total protein harvesting was performed by incubating the cells in lysis buffer (50 mM Tris pH 7.5; 1% NP40; 150 mM NaCl; 0.25% deoxycholic acid and 1 nM EGTA, supplemented with protease inhibitor cocktail (1:1000, Sigma) for 15 min on ice. Supernatant containing proteins was collected after centrifugation for 30 min at 13,000 g. Protein concentrations were measured by the Bradford protein assay (Bio-Rad, USA).

### Western-blot

A total of 50 μg protein extracts were separated on 10% SDS–polyacrylamide gel, for 2 h in 150 V and electro-blotted onto nitrocellulose membrane (Schleicher & Schuell Bioscience GmbH, USA), 250 mA, for 1.5 h. The membrane was blocked with 5% milk in PBS, 1 h at room temperature, followed by over-night incubation of the primary antibody at 4ºC. After 3 washes in TBS-T buffer, the membrane was incubated with a horseradish-peroxidase-conjugated (HRP) secondary antibody for 1 h, and after 3 additional washes, the electrochemiluminescence (ECL) reagents (Sigma) were incubated with the membrane for 2 min. The membrane was dried and placed on light sensitive film. The intensities of protein bands were quantified using ImageJ software.

### Statistical analyses

Comparisons of relative gene expression between study groups have been conducted by Mann–Whitney U-test, considering *p* < 0.05 as statistically significant. JMP Statistical Software package was used for statistical analysis.

## Results

### Transdifferentiation induces alterations in global DNA methylation profile

Aiming to analyze whether the pTF-induced TD process is associated with changes in the DNA methylation profile, we analyzed alterations in global DNA methylation during this process, using a methylation profiling microarray (Illumina Epic 850 K Methylation Array). We compared Illumina probes in pTF-treated human liver cells and in untreated and control-treated liver cells infected with adenoviral (Ad-β-gal) vector.

Different donors display distinct DNA methylation profiles. The effect of TD was analyzed in 2 different donors: donor A is a 9 year-old male, while donor B is an 18 year-old female. The two donors’ liver cells indeed displayed initial diversity in the state of methylation, as demonstrated in Additional file 1: Figure S1; out of 850,000 CpGs tested, 364,427 CpGs are significantly methylated in donors A and B (adjusted *P* value < 0.05). Out of them, 44,759 CpGs were hypomethylated and 144,341 hypermethylated with > 20% differences in donor B compared to donor A. These variances could be explained by the sex and age differences between the donors. (Additional file 1: Figure S1 for mean methylation of the 850 K probes in the two donors).

**Fig. 1:**
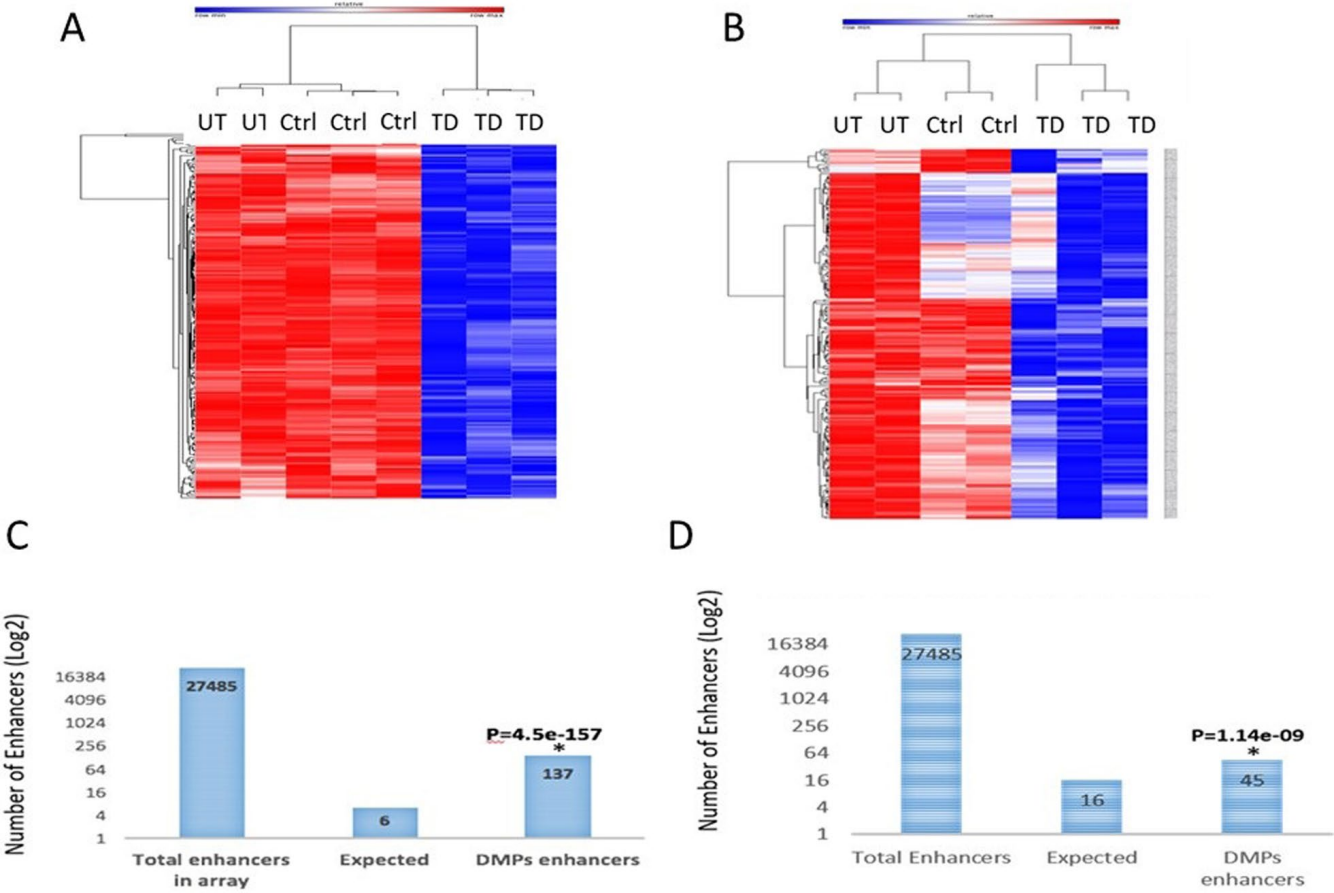
TD induces DNA de-methylation mainly at enhancer sites. Heat maps of the 192 CpGs differentially hypomethylated (< 10%) at day 6 post TD induction in donor A (**A**) and in donor B (**B**), comparing TD cells (TD) to un-treated liver cells (UT) or cells infected with adenoviral control (Ad-β-gal; Ctrl). Distribution of the hypomethylated DMPs in enhancers, in TD cells from donor A (**C**) and donor B (**D**). *p Value 4.5e−157 in C; *p* value = 1.14e−09 in D

Despite the differences in the basal DNA methylation between liver cells from the two donors, the direction of the DNA methylation alterations induced by the TD process was similar; in both cases TD was associated with hypomethylation (Fig. 1A–D) (Additional file 2: Table S1). Viral infection *per-se* had minimal effect on the DNA methylation profile, only 9 differentially methylated positions (DMPs) were significantly changed compared to the untreated cells after correction for multiple testing. However, 0.06% of the 850 K CpGs represented in Illumina chip were demethylated < −10%) in response to ectopic expression of pTFs in liver cells in both donors compared to the viral vector control (adjusted *P* value < 0.1) (192 DMPs in donor A and 191 CpGs in donor B, 10 CGs in Donor B and 0 CGs in donor A were hypermethylated in pTF-infected cells).

We analyzed the genomic distribution of the differentially methylated CGs, and noticed a very significant enrichment for CGs residing in enhancers (Fig. 1C, D), suggesting that the TD process causes demethylation of CGs at gene regulatory sites.

**Fig. 2:**
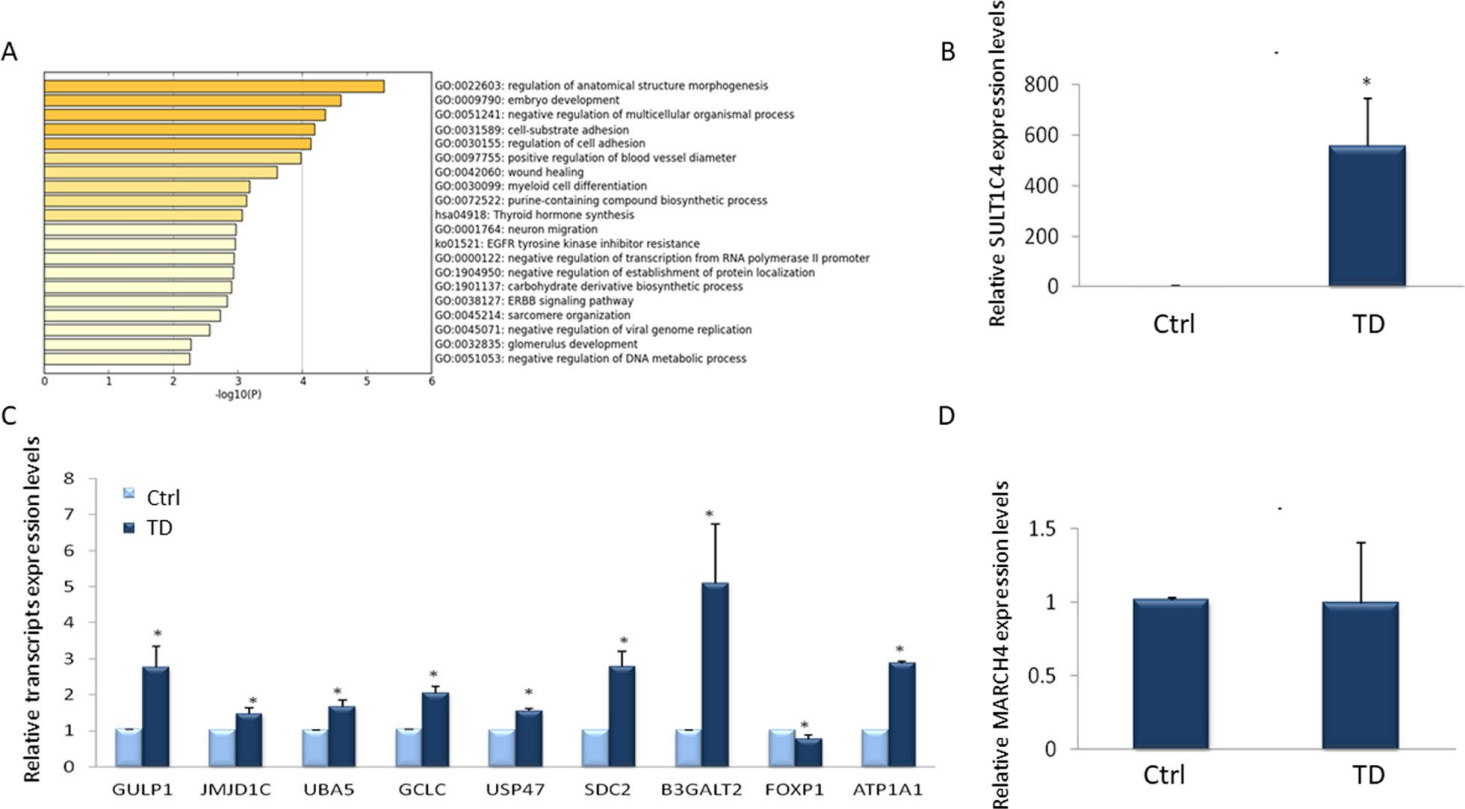
Activation of gene expression levels of demethylated genes in TD. **A** Functional analysis of DMPs with methylation differences (< −10%) (adj P value < 0.1). **B**–**D** Liver cells were induced to transdifferentiate for 6 days. The expression levels of genes with hypermethylated CpG’s (**B**, **C**) and genes with no change in DNA methylation (**D**) were measured by RT-qPCR. Results are presented as average ± SD, *n* = 6 different donors, *p* < 0.05

The selected DMPs were functionally classified using Metascape [19], as follows: the top enriched pathways pertain to regulation of anatomical structure morphogenesis, embryo development, negative regulation of multicellular structure morphogenesis, regulation of cell adhesion and cell-substrate adhesion (Fig. 2A).

### TD-induced DNA de-methylation is associated with increased gene expression

To analyze the functional relevance of the demethylation induced by the TD process, we measured the mRNA expression levels of genes regulated by the demethylated enhancers. Demethylation of enhancer regions is expected to cause increased gene expression of associated genes [20]. We performed RT-qPCR measurements of mRNA levels of selected genes, which displayed the highest extent of demethylation, and were located in proximity to the “transcription start site” (TSS), in RNA prepared from TD and control cells from 6 different donors. The results (Fig. 2B–D) demonstrate the induction of mRNA levels expressed by genes that were demethylated in TD-induced cells, except *FOXP1*. *SULT1C4* (Sulfotransferase Family 1C Member 4), which was demethylated in TD, showed a dramatic activation (Fig. 2B). We randomly selected *MARCH4* as a control gene since *MARCH4* methylation levels remained unchanged in TD cells, untreated cells, and viral control infected cells. As expected, *MARCH4* gene expression levels did not change during TD (Fig. 2D).

### Alterations in DNA methylation during TD is a progressive process

**Fig. 3:**
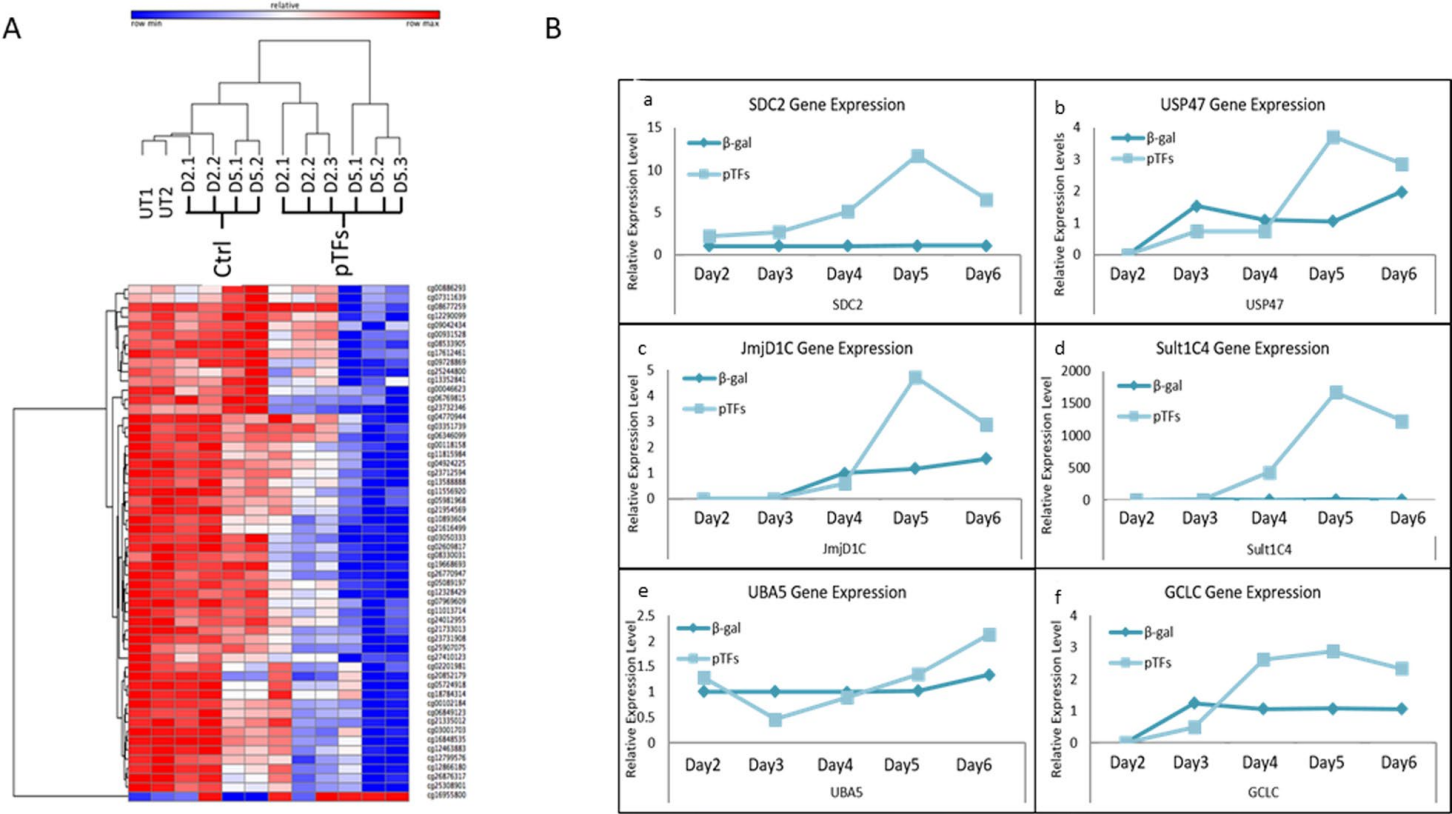
TD induces gradual CpG demethylation. **A** Heat map of top 52 differentially methylated CpGs with delta =  > 0.15 from donor B, at day 2 and day 6 after initiation of TD (adjusted *P* < 0.05) (Pearson linear correlation). **B** Liver cells were induced to transdifferentiate for 6 days. Gene expression analysis was performed 2–6 days post trans-differentiation of genes that were demethylated by the treatment: **A** *SDC1* (Syndecan 1), **B** *USP47* (Ubiquitin Specific Peptidase 47), **C** *JmjD1C*, (Jumonji Domain Containing 1C) **D** *SULT1C4*, **E** *UBA5* (Ubiquitin Like Modifier Activating Enzyme 5), and **F** *GCLC* (Glutamate-Cysteine Ligase Catalytic Subunit). Results are the average of RNA extracted from 3 different donors

The TD process is time-dependent (2–6 days post pTF infection) and is a consecutive and hierarchical process, as demonstrated by Berneman-Zeitouni et al. [6]. We analyzed the time course of the DNA de-methylation process at 2 and 5 days,and the temporal activation of pancreatic gene expression. The results show progressive demethylation from day 2 to day 5 (Fig. 3A). Similarly, gene expression is progressively induced from day 2 to day 5 (Fig. 3B).

### Induction of DNA de-methylation promotes the trans-differentiation process

Our data suggest that transdifferentiation is associated with DNA demethylation. To determine whether DNA demethylation might be playing a causal role in the TD process, we analyzed the effect that DNA methylation blockade by either shRNA DNMT1 or by pharmacological inhibition has on the TD process, as DNMT1 is responsible for the maintenance of existing methylation patterns across DNA replication [21].

**Fig. 4:**
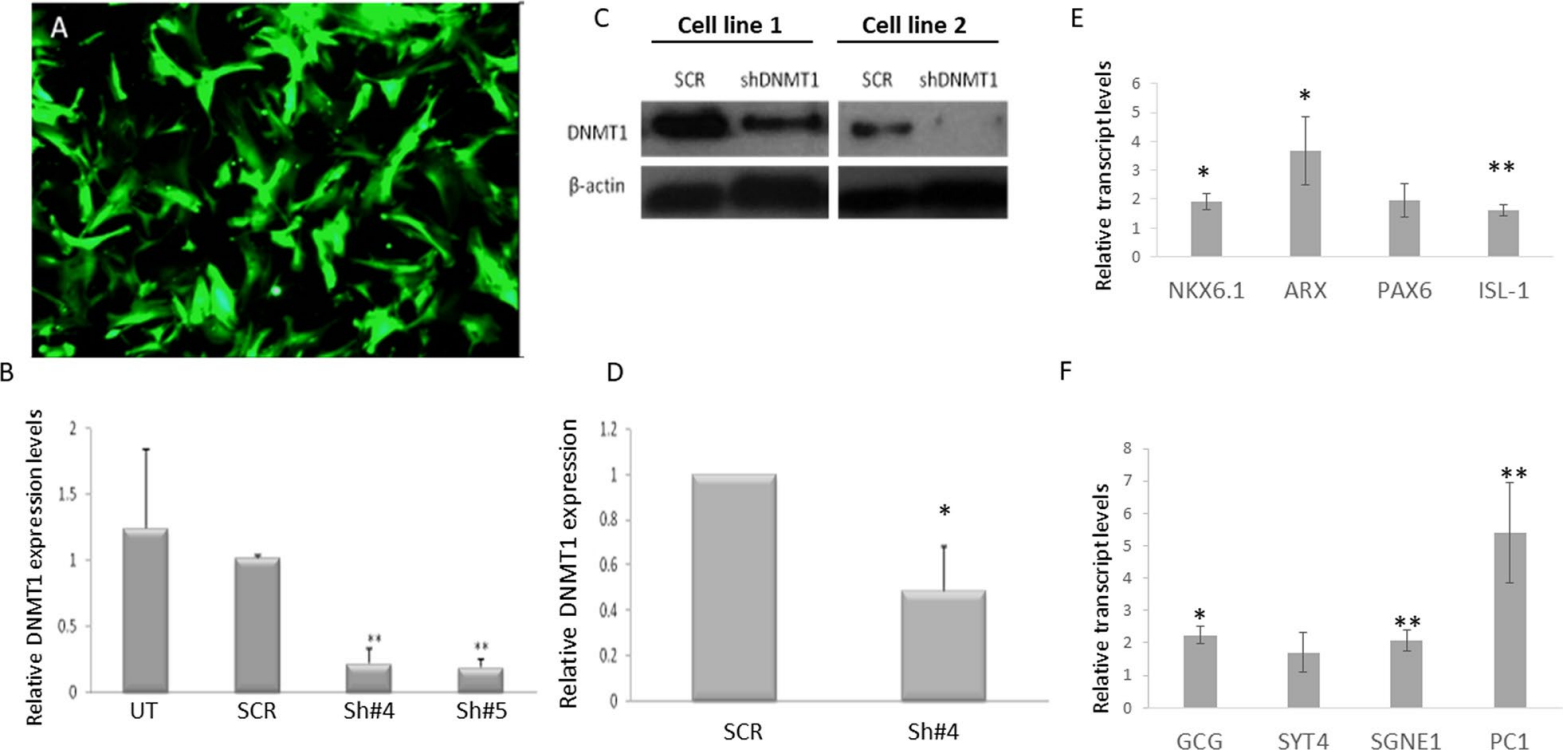
Downregulation of DNMT1 expression promotes pTFs induced TD: DNMT1 expression was knocked-down using specific shRNAs (sh#4 and sh#5) delivered by GFP-expressing lentiviruses, as described in materials and methods: lentivirus infection efficiency was measured by **A** GFP expression of treated cells at 2 weeks post lentiviral infection. **B** mRNA levels of DNMT1 at 2 weeks post lentiviral infection. *N* = 8 *p* < 0.000003. **C** Western blot analysis of DNMT1 protein levels in 2 selected cell lines at 2 weeks post infection. **D** Protein level quantification of DNMT1 at 2 weeks post lentiviral infection, compared to scrambled shRNA delivery. *N* = 4, *p* < 0.0006. Stable clones of DNMT1 knocked down liver cells were transdifferentiated by ectopic expression of pTFs. The expression levels of pancreatic specific TFs (**E**) and pancreatic specific genes (**F**) was measured 6 days post TD induction by RT-qPCR. Results are presented as average ± SD, *n* = 4 different donors, *p < 0.01, **p < 0.05

DNMT1 knockdown was performed in primary human liver cultures using lentivirus-mediated specific DNMT1 shRNA delivery. Following highly efficient infection of the cells (Fig. 4A) DNMT1 was reduced at the mRNA (Fig. 4B) and protein levels (Fig. 4C and quantification in 4D) at 1 week post infection. The DNMT1 knocked down (KD) cells were submitted to the TD process, and the efficiency of the process was analyzed and compared to the scramble shRNA (SCR)-treated control clones by measuring expression of pancreatic-specific genes, in response to ectopic pTFs expression. DNMT1 KD clones exhibited 5.5–1.5-fold increased expression of pancreatic-specific genes compared to scramble shRNA-treated control cells, (Fig. 4E, F).

**Fig. 5:**
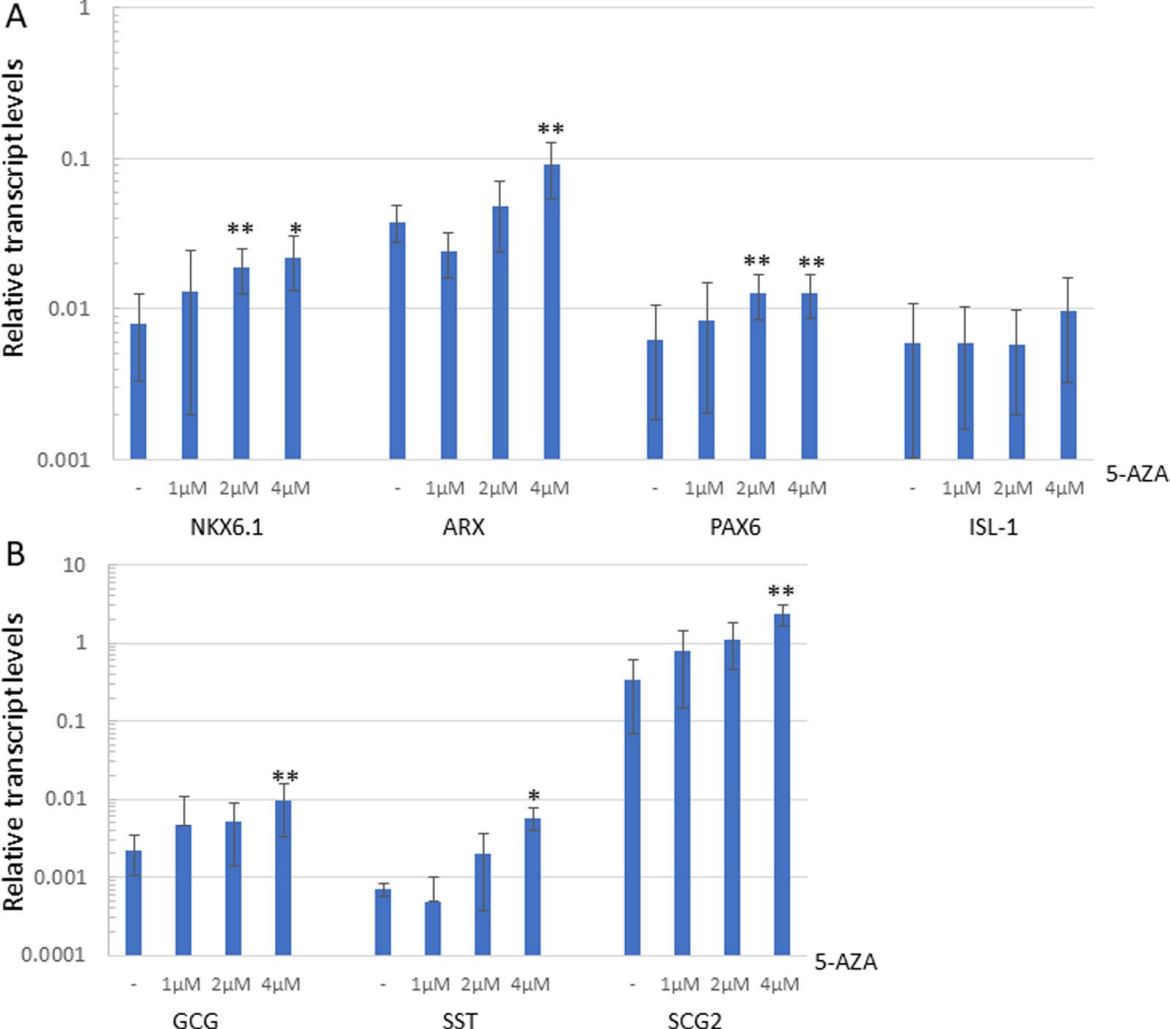
DNA demethylation agent 5-AZA promotes pTF-induced TD: liver cells were treated by increasing amounts of 5-AZA (0–4 µM), 48 h before induction of trans-differentiation by ectopic pTF expression. The expression levels of pancreatic specific transcription factors (**A**) and pancreatic specific genes (**B**) was measured 6 days post TD induction by RT-qPCR. Results are presented as average ± SD, *n* = 6 different donors, *p < 0.01, **p < 0.05

To further confirm that DNA demethylation can induce pancreatic genes in liver cells, we treated the cells with the DNA methylation inhibitor 5-azacytidine (5-AZA). Liver-derived cells were treated with increased concentration of 5-AZA (0–4 µM), together with the induction of the TD process by the recombinant adenovirus-delivered pTFs. Our data demonstrate that high concentration of 5-AZA (2–4 µM) significantly increased the expression of pancreatic-specific genes in pTFinduced TD cells (Fig. 5, 5A-induced pancreatic transcription factors, 5B-induction of pancreatic specific genes expression).

### TD-prone human liver cells are characterized by a basal state of DNA-hypomethylation

**Fig. 6:**
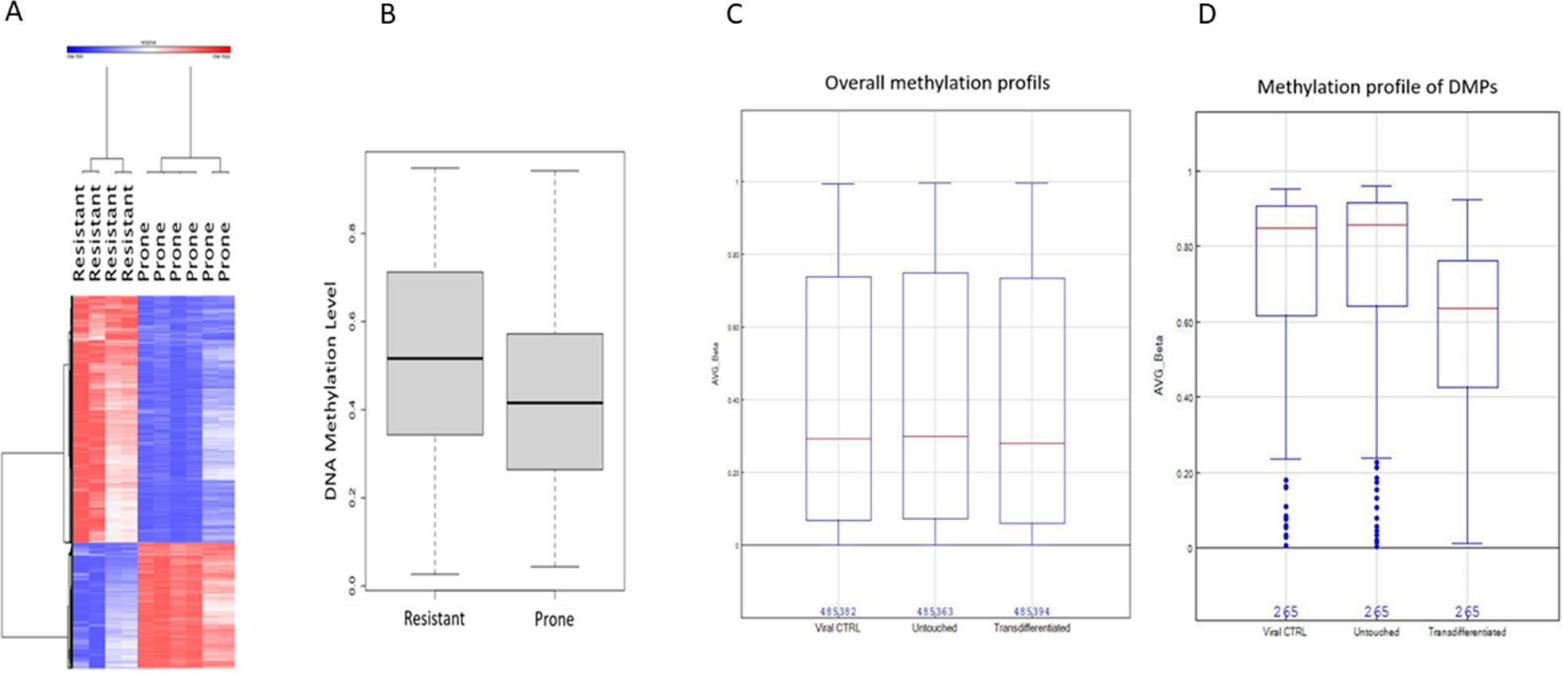
TD-prone cells display basal DNA hypomethylation compared to TD-resistant liver cells and further undergo demethylation upon pTF-induced TD **A** Heat map of 6017 CpGs with over 20% differences in resistant (*n* = 4) and prone cells (*n* = 6), adjP < 0.05. **B** Boxplot display of 6017 over 20% DMPs in resistant (*n* = 4) vs. prone (*n* = 6) human liver primary cell lines, adj*P* < 0.05. TD-prone cells underwent the TD protocol, and average global methylation across all probes in Infinium HumanMethylation450 BeadChip Kit was determined (Illumina). **C** Overall methylation profile of viral control (β-gal) infected cells, untreated cells (untouched), and TD cells. Red lines indicate the average methylation level. **D** Methylation profile of altered meCgGs. Compared to untreated cells, 265 CpG’s methylation levels were significantly altered in TD cells

Cohen et al. [7] suggested that cells originating from hepatic pericentral populations possess an innate propensity for reprogramming into endocrine pancreas. These cells are characterized by an active Wnt signaling pathway that is obligatory but insufficient to promote pTF-induced TD [7]. The same study suggested that permissive chromatin organization and active Wnt signaling are the two requirements that are necessary and sufficient for the reprogramming-predisposition of liver cells. Since our present data also suggest a role for DNA hypomethylation in liver-to-pancreas TD, we sought to compare the basal DNA methylation status of TD-prone cells to that of TD-resistant liver cells taken from the same human liver donor. Figure 6A suggests that indeed, the TD-prone cells (derived from multiple donors) are further characterized by DNA-hypomethylation compared TD-resistant cells (Additional file 3: Table S2).

Taken together, these data suggest that the initial state of DNA methylation could contribute to cellular TD-predisposition, and that TD-prone liver cells are a priori characterized by reduced DNA methylation.

The Wnt signaling activation, HDAC inhibition and DNA hypomethylation have concerted effect on promoting pTF-induced TD efficiency.

**Fig. 7:**
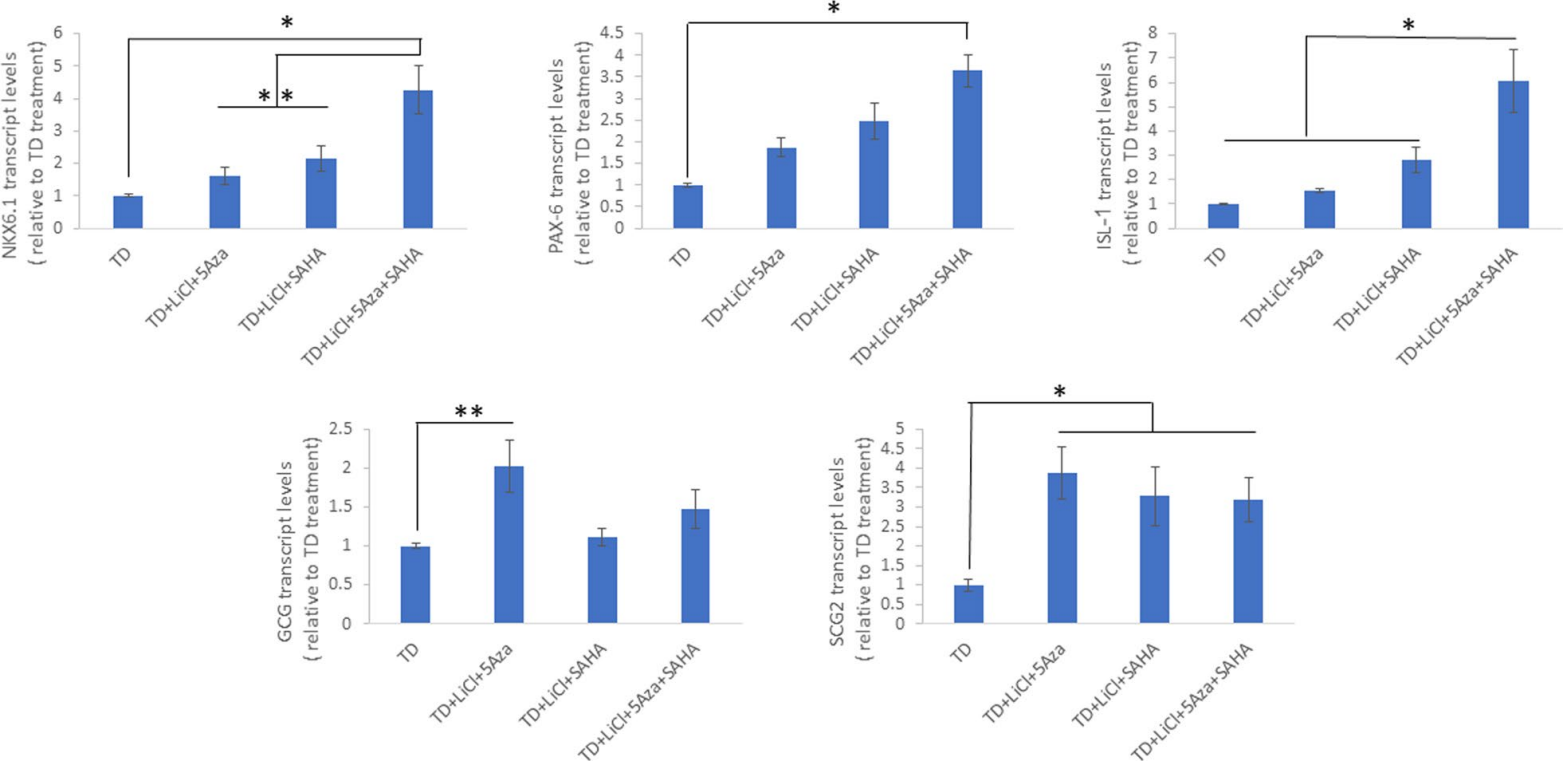
Significant increase in TD efficiency occurs upon combination of pTFs with epigenetic modifying agents. Liver cells were treated with a combination of soluble factors, as described in the materials and methods section, and infected 48 h later with pTFs. Expression of the pTFs NKX6.1,PAX6, and ISL-1 as well as the pancreatic specific genes GCG, SST and SCG2was analyzed by RT-qPCR. Results are presented as average ± SD, *n* = 3 different donors, *p < 0.01, **p < 0.05 related to TD alone, ^##^*p* < 0.01, ^#^*p* < 0.05 related to TD + LiCl + 5-AZA + SAHA

To further analyze the relative contribution of DNA methylation to the epigenetic modifications which characterize TD-propensity of liver cells towards the pancreatic lineage, we analyzed the combined effects of epigenetic modulators: histone deacetylase inhibitor SAHA, DNA methylation inhibitor 5-AZA and Wnt signaling activator (LiCl) on the TD-efficiency of liver cells (Fig. 7).

The expression of the pTFs (NKX 6.1, Pax-6 and ISL-1) was significantly increased upon the concerted treatment by all three epigenetic modulators. However, the expression of the downstream pancreatic genes, Glucagon and SCG2 was maximally increased only upon LiCl and 5-AZA treatment alone. The chromatin modifier (SAHA) did not further increase these genes’ expression.

Taken together, our data suggest a critical role for permissive chromatin organization, active Wnt signaling and DNA hypomethylation on promoting pTFs induced liver to pancreas TD efficiency. These three factors seem pivotal to the reprogramming capacity of liver cells along the endocrine pancreatic phenotype.

## Discussion

The present study suggests that the pTF-induced developmental process plays a proactive role in inducing DNA hypomethylation in the hepatic host cells. The ectopic transcription factors induce a gradual process of DNA demethylation which initiates as soon as two days post viral infection. Most demethylated differentially methylated regions (DPMs) are located at enhancer regions, implying that TD affects the methylation state at gene regulatory sites, which is indeed reflected in increased gene expression.

We did not find changes in DNA methylation of pancreatic or hepatic targets, among the genes that display an altered methylation profile. This may suggest that during the TD process, the ectopic pTFs may indirectly affect expression of pancreatic and hepatic-specific genes by demethylation of regulatory genes which prime the epigenetic landscape alterations needed for alternate pancreatic lineage induction.

Consistent with the idea that DNA demethylation is required for TD, when separating human liver cells from multiple human donors according to their TD-propensity [7], TD-resistant cells displayed a higher level of DNA methylation compared to that of TD-prone cells derived from the same donor (Fig. 6). Thus, although pTFs can induce demethylation, they could do so only on an epigenomic matrix that becomes permissive to their action by demethylation of other positions in the genome. In line with our findings, Wang et al. [22] suggested TD-resistant cells to be self-renewing cells that contribute to homeostatic maintenance of hepatocytes by differentiating into and replacing other hepatocytes along the hepatic lobule in the normal liver.

Consistent with the idea that DNA demethylation is required for trans- differentiation, DMNT1 inhibition or 5-AZA treatment promotes pTF-inducedTD and increases its efficiency, possibly either by demethylating critical positions in the genome that could not be demethylated by the pTFs on their own and are nevertheless required for differentiation, or by enhancing the demethylation induced by pTFs. DNA demethylation agents promoted pTF-induced TD both alone and in concert with additional chromatin modifying agents and Wnt intracellular signaling activators, such as LiCl [7].

Our previous studies suggested that PDX-1 plays a dual role in the process of liver-to-pancreas trans-differentiation; while repressing the hepatic repertoire of gene expression in each liver cell, it activates the pancreatic lineage only in predisposed liver cells [3]. We did not find any increased DNA methylation at PDX-1 known recognition sites on hepatic genes that were silenced during TD [3], even one month post TD induction (data not presented). This may potentially suggest that the inhibition of the host repertoire of gene expression by PDX-1 is not mediated by DNA methylation of its direct target genes. This is further strengthened by the fact that transdifferentiation is associated mainly by induced hypomethylation but not hypermethylation. Other epigenetic modulations such as the polycomb-mediated gene silencing which is known to occur during lineage commitment [23], demethylation of suppressor genes, microRNAs should be further analyzed.

Taken together, our study suggests that TD *per-se* induces alteration in global DNA methylation profiles in a progressive manner, starting sooner than two days after the pTFs’ ectopic expression, which, in turn, promotes the activation of induced pancreatic gene expression. The concerted effect of epigenetic modulators may increase chromatin permissiveness for pTF action, thus promoting the efficiency of the developmental process which activates pancreatic lineage and function in liver cells. A limitation is that the in vitro study results provide mechanistic findings and accordingly identify soluble factors that could increase the TD process’ efficiency. The implementation of these results in improving the therapeutic consequences of this potential cell therapy approach for diabetes should be further studied.

In detail our study suggests the followings:

- The TD process is associated with increased global DNA demethylation, mainly at gene regulatory elements and with increased expression of these genes.
- Both DNA hypomethylation, as well as activation of gene expression are progressive, initiating about 2 days after ectopic expression of pTFs.
- Inhibition of DNA methylation promotes the pancreatic transcription factor-induced liver-to-pancreas TD process.
- DNA methylation is not directly involved in the repression of the hepatic repertoire of gene expression by PDX-1.
- Human liver cells that are predisposed to undergo pTF-induced TD are also characterized by DNA hypomethylation, compared to the entire cell population.
- HDAC inhibition and DNA demethylation work in concert with Wnt signaling activation to promote pTF-induced TD efficiency

## Future perspective

Liver cell reprogramming into insulin producing cells (IPCs) is a promising approach toward autologous regenerative therapy for diabetes patients. Cell differentiation fate can be reprogrammed by overexpression of selected transcription factors, usually a subset of those required for normal development of the relevant cell type. The current manuscript presents a basic science conceptual study that continues to analyze our pioneer research on transcription factors induced liver to pancreas transdifferentiation [24]. Numerous studies suggested that the ectopic pTFs serve as a short-term trigger to an irreversible reprogramming process, in which the sequence of the genetic information remains unaffected, but the identity of hundreds and thousands of expressed genes is irreversibly altered [2, 3, 7, 24, 25]. It has been postulated that transdifferentiation is an epigenetic process that involves the activation of otherwise silent genes, while silencing many of the originally expressed genes [3]. Subsequently altering the developmental fate and function of adult cells and tissues the epigenetic events that mediate this process are only poorly understood and only partially studied.

This manuscript is a continuation of our previous pioneer studies in the field of liver to pancreas transdifferentiation showing for the first time that the pancreatic transcription factors used for inducing liver to pancreas TD induce also a DNA de-methylation and the induced hypomethylation is indeed relevant to the TD process efficiency. Both the activation of the alternate pancreatic repertoire [6] and the induced hypomethylation are progressive processes, with a similar time frame of induction. However, in order to follow process and mechanisms in a lab setting, we are using high concentrations of the analyzed soluble material (SAHA, 5AZA, DNMT1 knockdown). Such treatments affect the cells viability. Therefore, we are using “read-out” analyses such as gene expression. Other functional assays both *in vivo* and *in vitro* are technically challenging.

## Supplementary Information

**Additional file 1**. **Figure S1** Differences in DNA methylation status between two different donors: methylation profile of 364,427 significantly differential methylated CpGs in donor A and donor B; 44,759 CpGs are significantly demethylated and 144,341 are hypermethyalted more than 20% in donor B compared to donor A, adjusted *P* value < 0.05.

**Additional file 2**. **Table S1.** TD induces hypomethylation.

**Additional file 3**. **Table S2.** TD-prone cells compared to TD-resistant cells.

## Data Availability

Not applicable.

## Abbreviations

TD: Transdifferentiation
pTFs: Transcription factors
HDACs: Histone deacetylases
HDACi: Histone deacetylase inhibitor
DNMT: DNA methyltransferases
SAHA: Suberoylanilide hydroxamic acid
5-AZA: 5-Azacytidine
LiCl: Lithium chloride
IPCs: Insulin producing cells
TSS: Transcription start site
DPMs: Differentially methylated regions
