# Supplementary figures and images for: The role of DNA demethylation in liver to pancreas transdifferentiation
Source: Stem Cell Res Ther. 2022 Sep 16;13:476. doi: 10.1186/s13287-022-03159-6 (PMC9482206; doi:10.1186/s13287-022-03159-6)

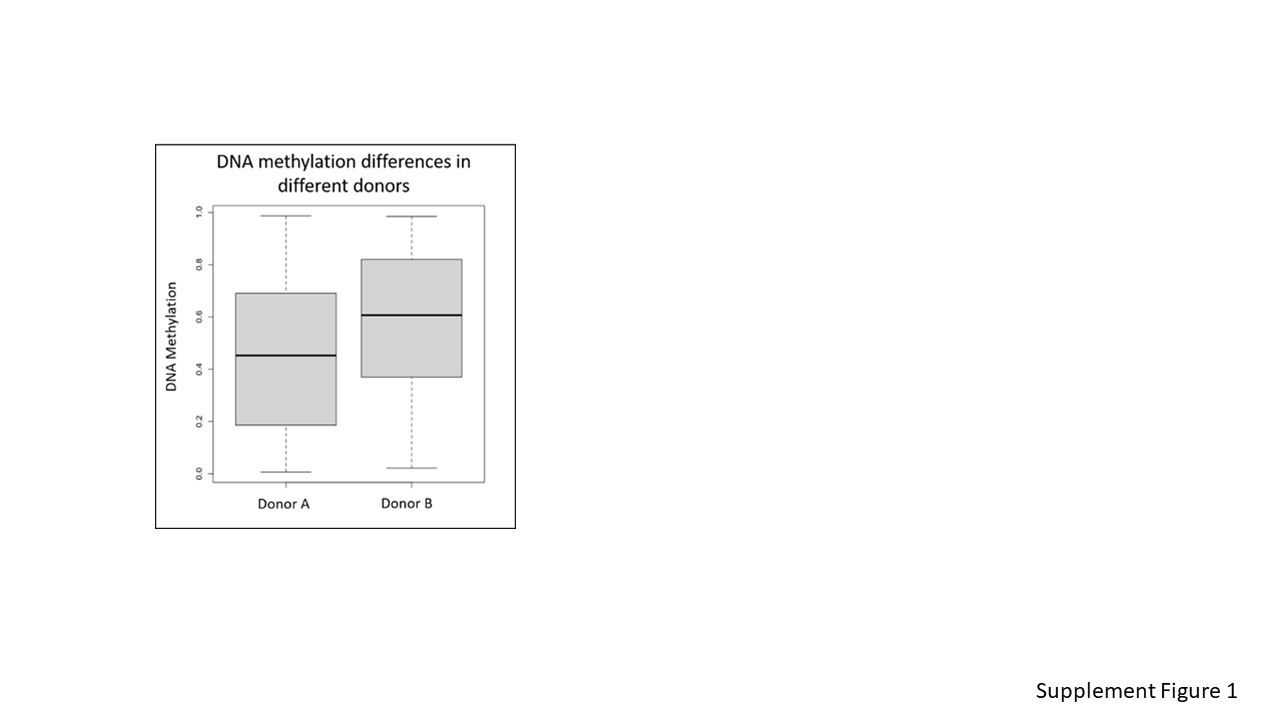

Supplement: Supplementary file 1 — Additional file 1. Figure S1 Differences in DNA methylation status between two different donors: methylation profile of 364,427 significantly differential methylated CpGs in donor A and donor B; 44,759 CpGs are significantly demethylated and 144,341 are hypermethyalted more than 20% in donor B compared to donor A, adjusted P value < 0.05. [file 13287_2022_3159_MOESM1_ESM.tif]
